# Aptamer technology for tracking cells’ status & function

**DOI:** 10.1186/2052-8426-2-33

**Published:** 2014-10-27

**Authors:** Christian Wiraja, David Yeo, Daniel Lio, Louai Labanieh, Mengrou Lu, Weian Zhao, Chenjie Xu

**Affiliations:** Division of Bioengineering, School of Chemical and Biomedical Engineering, Nanyang Technological University, 70 Nanyang Drive, Singapore, 637457 Singapore; Department of Pharmaceutical Sciences, Sue and Bill Gross Stem Cell Research Center, Chao Family Comprehensive Cancer Center, University of California Irvine, Irvine, CA 92697 USA; Department of Biomedical Engineering, Edwards Lifesciences Center for Advanced Cardiovascular Technology, University of California Irvine, Irvine, CA 92697 USA

**Keywords:** Aptamer, Biosensor, Contrast agent, Cell tracking, Stem cells, Immune cells, Cancer cells

## Abstract

In fields such as cancer biology and regenerative medicine, obtaining information regarding cell bio-distribution, tropism, status, and other cellular functions are highly desired. Understanding cancer behaviors including metastasis is important for developing effective cancer treatments, while assessing the fate of therapeutic cells following implantation is critical to validate the efficacy and efficiency of the therapy. For visualization purposes with medical imaging modalities (e.g. magnetic resonance imaging), cells can be labeled with contrast agents (e.g. iron-oxide nanoparticles), which allows their identification from the surrounding environment. Despite the success of revealing cell biodistribution *in vivo*, most of the existing agents do not provide information about the status and functions of cells following transplantation. The emergence of aptamers, single-stranded RNA or DNA oligonucleotides of 15 to 60 bases in length, is a promising solution to address this need. When aptamers bind specifically to their cognate molecules, they undergo conformational changes which can be transduced into a change of imaging contrast (e.g. optical, magnetic resonance). Thus by monitoring this signal change, researchers can obtain information about the expression of the target molecules (e.g. mRNA, surface markers, cell metabolites), which offer clues regarding cell status/function in a non-invasive manner. In this review, we summarize recent efforts to utilize aptamers as biosensors for monitoring the status and function of transplanted cells. We focus on cancer cell tracking for cancer study, stem cell tracking for regenerative medicine, and immune cell (e.g. dendritic cells) tracking for immune therapy.

## Introduction to cell tracking

Over the past decade, the field of cell tracking, which includes monitoring cellular location, function and behavior in real time through various imaging modalities, has experienced rapid progression. The successful clinical translation of therapeutics have motivated researchers to seek a comprehensive understanding of the *in vivo* fate of transplanted cells [1, 2]. For instance, tracking cancer cells in their native environment can yield data on their biodistribution, tropism, status and functions (e.g. metastasis), which can significantly impact the success of cancer therapy [3–5]. Meanwhile, revealing the fate and functions of therapeutic cells following their implantation can help optimize the procedure of cellular therapy (e.g. dosage, injection frequency, and administration protocol) [6].

In both preclinical and clinical studies, cells can be monitored and tracked through imaging modalities such as: optical imaging, positron emission tomography (PET)/single photon emission computed tomography (SPECT), X-ray computed tomography (CT), and magnetic resonance imaging (MRI). Typically, cells of interest are labeled with contrast agents that provide detectable signals to distinguish them from bystander cells. For example in optical imaging, fluorescent/bioluminescent molecules and nanoparticles are used as contrast agents [7, 8]. On the other hand, PET/SPECT employs radio-isotope labeling agents such as ^18^F-FDG [9, 10]. Agents with high X-ray absoption properties (e.g. Omnipaque) meanwhile, are used to label cells for X-ray imaging and CT [11]. Lastly, MRI utilizes gadolinium or iron oxide nanoparticles to modify the magnetic relaxation time of the selected tissue [12, 13]. Although these contrast agents have greatly assisted researchers to visualize the shape, morphology and motion of cells, tissues, and organs, few have the ability to specifically reveal the status and function of cells at a high spatiotemporal resolution. In addition, they generally suffer from significant uptake and transfer to non-target cells [14–16].

Ideally, contrast agents for cell tracking should efficiently label cells of interest, persist within the cells for a period of time with minimal transfer to bystanders, and provide a detectable change in signal to reflect changes in cell status and/or function.

## Review

### Aptamer-based biosensors

Aptamers are single-stranded RNA or DNA oligonucleotides usually 15 to 60 bases in length that can bind specifically to target molecules. Typically, aptamers can be generated from a selection process termed as SELEX (systematic evolution of ligands by exponential enrichment) [17, 18]. In SELEX, an initial library consisting of >10^13^ random oligonucleotides is enriched by an iterative elimination and PCR process to selectively amplify sequences possessing high affinity to the pre-determined target.

**Figure 1:**
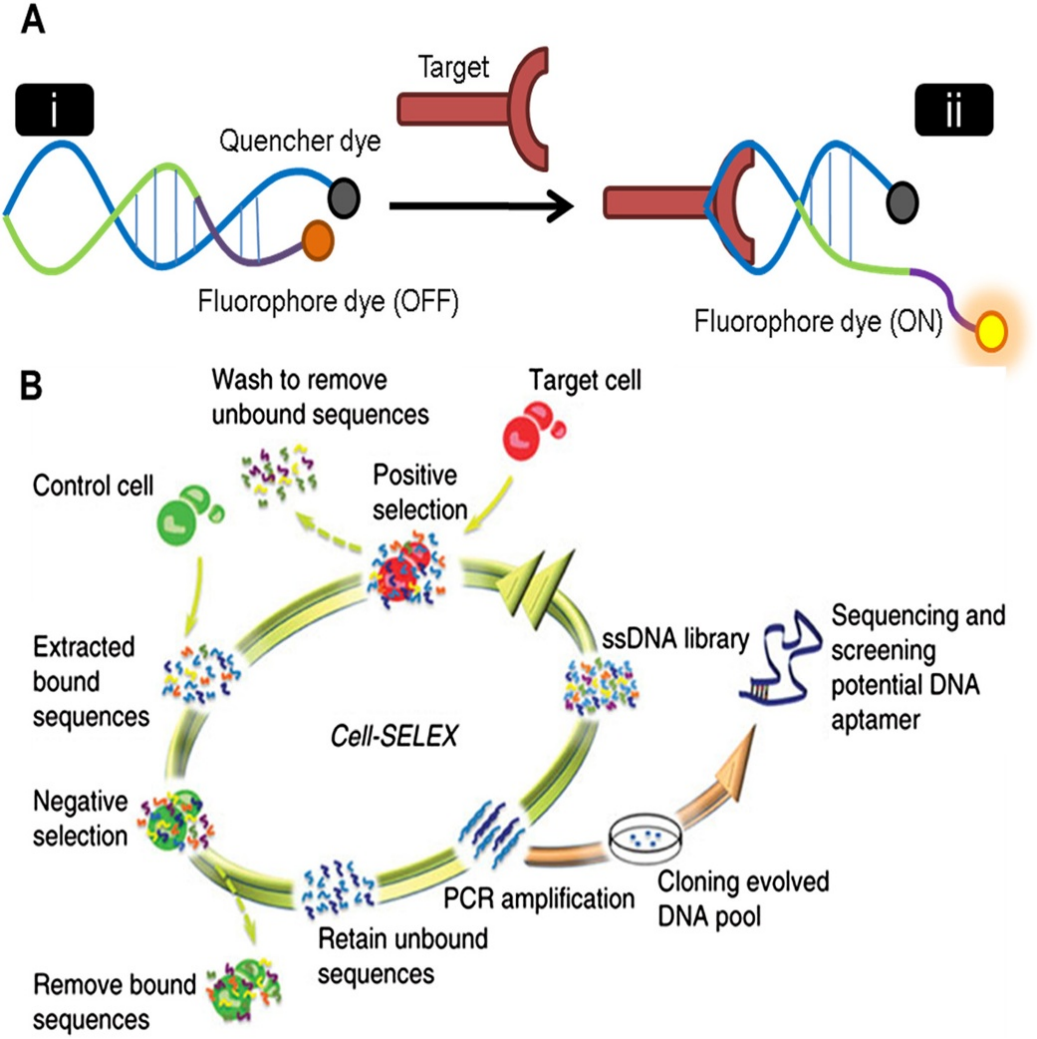
**Mechanism and selection process of aptamer probes. A)** Hybridization of aptamer probes with their target molecule involves a structural change (from i to ii), which triggers fluorescent signal restoration due to the increased distance between the fluorophore and the quencher. **B)** Selection steps within one cycle of cell-SELEX. Briefly, a library of single-stranded sequences is incubated with target cells. Following the washing procedures, negative selection is done to remove sequences that bind non-specifically. Subsequently, the resulting sequences are PCR-amplified before proceeding to the next cycle. Part B is adapted with permission from ref. [38]. Sefah K, Shangguan D, Xiong X, O’Donoghue MB, Tan W: Development of DNA aptamers using Cell-SELEX. Nature protocols 2010, 5:1169–1185. Copyright 2010 by Nature Publishing Group.

With the versatility of target molecules for the SELEX process, a wide range of aptamer applications have been developed, such as immobilized sensing molecules (aptasensors), since its introduction in 1990 [17]. For instance, aptamers have been conjugated on the surface of gold nanoparticles (AuNP) to recognize and detect the presence of small analytes including K^+^, ATP, and cocaine [19–21], as well as larger proteins like thrombin and platelet-derived growth factors (PDGF) [22, 23]. These aptasensors rely on the highly specific, structure-switching ability of aptamers; they undergo drastic secondary or tertiary folding from their initial conformation upon binding with their target molecules [24]. By labeling aptamers with quencher and fluorophore dyes at their 5’ and 3’ ends, a target binding event, which causes a displacement of the two dyes can be transduced to a change in fluorescent signal as a result of Förster resonance energy transfer (FRET) principles (Figure 1A) [25].

While the application of SELEX for whole-cells target (cell-SELEX) is relatively new, it has progressed rapidly over the past decade. In comparison to other targeting ligands such as antibodies, aptamers exhibit several advantages. Firstly, the synthesis of aptamer is an entirely chemical process that can be scaled up with consistency and used to incorporate a diverse range of functional moieties [26–28]. In addition when compared to antibodies, aptamer probes are low in immunogenicity and considerably stable in a wide range of pH (4–9), temperature, and organic solvents [29–31]. Furthermore, the molecular-level resolution of aptamers can be utilized to transduce signal changes related to cellular status. By employing aptamers targeted at a specific cell surface marker for example, the up/down-regulation of that particular marker can be monitored, as a mean to evaluate cellular functionality [32–34]. As will be elaborated later, this approach has been successfully utilized to evaluate embryonic stem cell (ESC) differentiation processes [35]. Meanwhile, the high specificity and low dissociation constant (K_d_) (in the range of nano to pico-molar) of aptamers help to minimize the risk of agent transfer to non-target cells [36]. Taken together, these properties make aptamers an excellent tool to accomplish ideal cell tracking (i.e. complete monitoring of cellular biodistribution, status and functions), for both diseased cells (e.g. cancer cells) and therapeutic cells (immune cells & stem cells).

Lastly, it is also noteworthy that cell-SELEX (Figure 1B) does not require prior knowledge regarding the surface signature of the target cells. Instead, the selection of aptamers involves repetitive elimination of sequences that bind to non-target cells [37–39]. For example to screen aptamers for targeting human T cell lymphoblast-like cell line CCRF-CEM cells, a sequence library was continuously enriched for sequences that bind to CCRF-CEM cells but not Ramos cells. The library enrichment was quantitatively evaluated over the different cycles of incubation and selection steps by monitoring the fluorescence of CCRF-CEM cells through flow cytometry [40].

### Aptamers for cell tracking

Early applications of aptamers for cell tracking were focused on diseased cells. Particularly, scientists have utilized aptamers as “molecular-level beacons” to better understand the behavior of cancer cells in vivo [40, 41]. By selecting aptamers that recognize cancer antigens and coupling them with a contrast agent, such as quantum dots (QDs), fluorescent dyes, or iron oxide nanoparticles (IO-NPs) [28, 42, 43], cancer cells can be non-invasively monitored for their location, growth progression, metastatic migration, etc. [33, 44, 45]. As compared to end-point analysis (e.g. histology of sample biopsies) which is a destructive process [46, 47], aptamers provide real-time tracking, which more accurately represents the dynamic *in vivo* microenvironment, while minimizing the number of preclinical experimental subjects [48, 49]. Given the recent advancements in regenerative medicine, aptamers are beginning to be incorporated in the development of cellular therapy and tissue engineering. In such applications, aptamers are potentially beneficial not only to reveal the biodistribution and tropism of cells, but also to report their successful utilization *in vivo* (i.e. achieving desirable therapeutic effects and functional tissues) [50].

**Figure 2:**
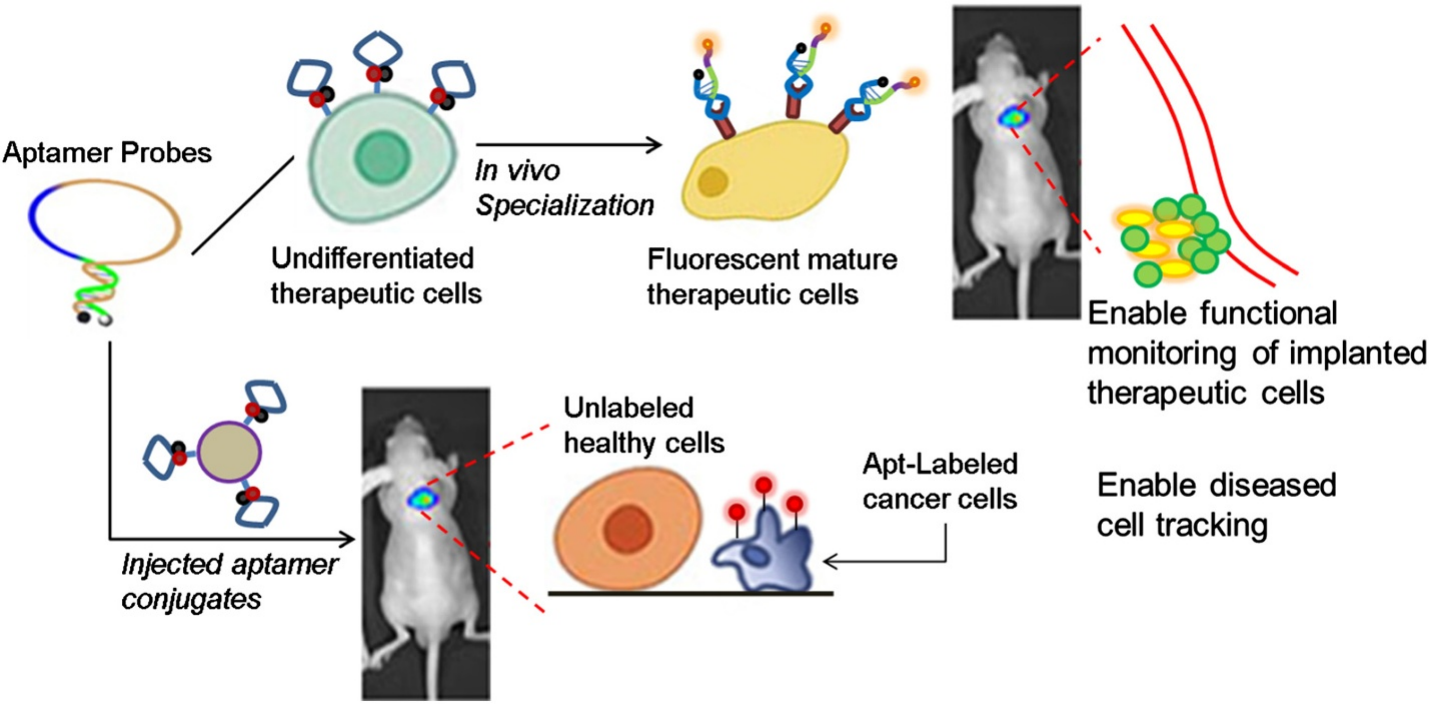
**Overview of aptamer probes application for cell tracking.** Aptamers can be attached to therapeutic cells ex-vivo, to track their bio-distribution, tropism and functions (e.g. differentiation for stem cell) following their implantation. Additionally, aptamer conjugates can be systemically introduced to specifically locate and longitudinally monitor diseased cells (e.g. metastatic cancer cells).

Below, we first describe the applications of aptamers for tracking cancer cells. Subsequently, aptamer applications for two important cell types in the field of regenerative medicine (stem cells and immune cells) will be discussed in detail. An overview of the aptamer-based cell tracking applications can be found in Figure 2.

### Aptamers for tracking cancer cells

Efforts using aptamer-based cancer cell tracking can be grouped under the different imaging modalities, including optical, PET/SPECT, X-ray/CT, and MRI.

Optical imaging is perhaps the most common means of aptamer tracking. Its distinctive properties such as easy accessibility, low instrumentation cost, high resolution (allowing visualization of single cells) and good spatiotemporal sensitivity, promote utilization in many tracking applications. Furthermore, the recent advancements of multi-photon and intra-vital microscopy and optical probes in the near-infrared region (NIR) (700-900 nm) have permitted *in vivo* optical imaging on small animals or for near-surface observation [51–53].

**Figure 3:**
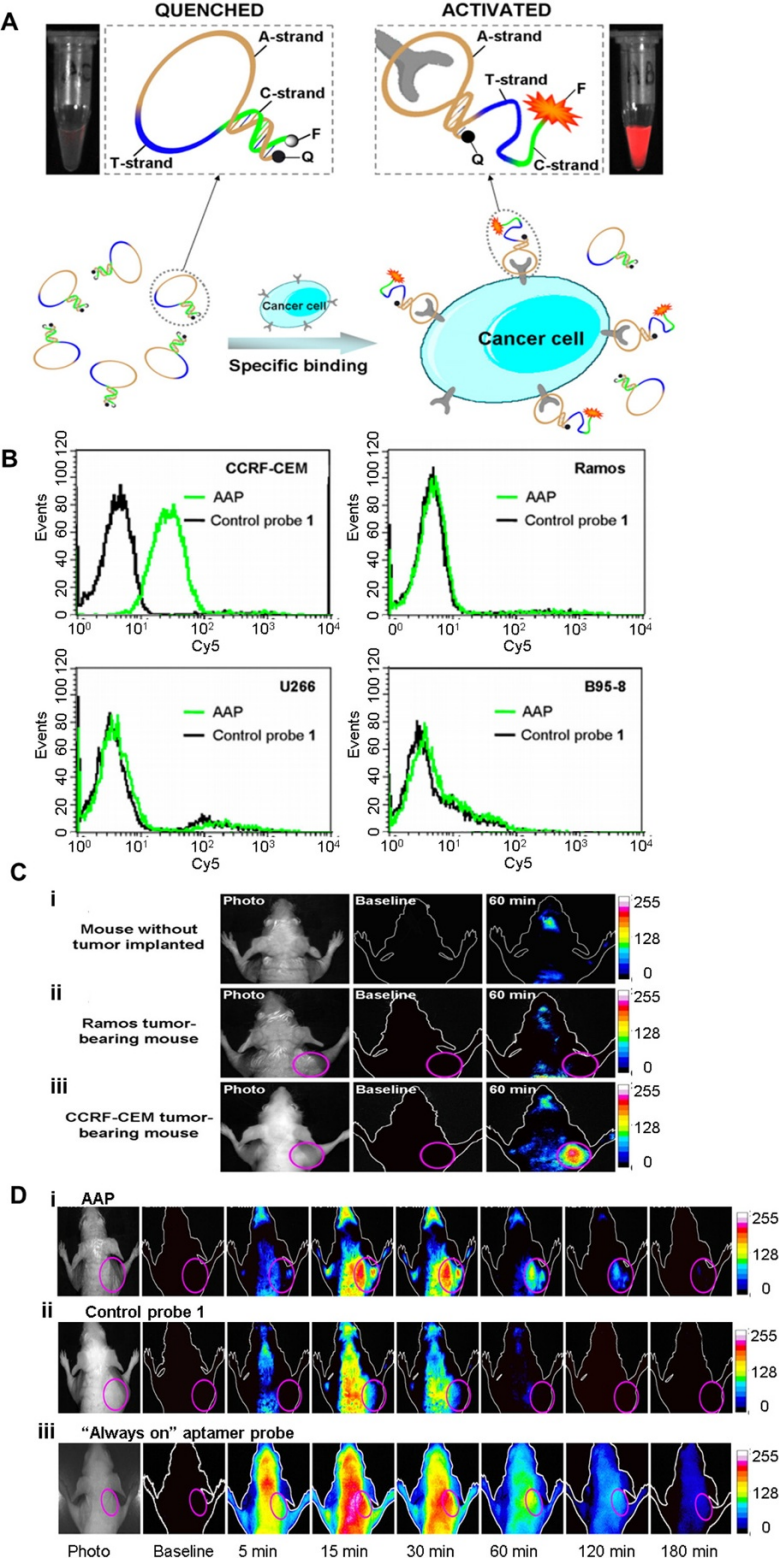
**Activatable aptamer probe (AAP) for** ***in vivo*** **cancer tracking. A)** Schematic showing the mechanism of AAP recognition against CCRF-CEM cells. Upon specific binding, the Sgc8 aptamer-containing AAP exhibited conformation alteration, which resulted in fluorescent restoration. **B)** Flow cytometry data shows the specificity of AAP binding towards CCRF-CEM cells. As comparison, the signal from control probes with no affinity was shown. **C)** Specific *in vivo* CCRF-CEM tumor imaging with AAP. One hour following their intravenous injection, AAP accumulated pre-dominantly on CCRF-CEM tumor sites (labeled with pink circle), but not to control Ramos tumors. **D)** Longitudinal imaging of CCRF-CEM tumor with AAP revealed clear tumor signal between 5 to 120 minutes post-intravenous injection. In comparison, control and “always-on” probe did not show specific fluorescent signal at tumor site. Adapted with permission from ref. [55]. Shi H, He X, Wang K, Wu X, Ye X, Guo Q, Tan W, Qing Z, Yang X, Zhou B: Activatable aptamer probe for contrast-enhanced in vivo cancer imaging based on cell membrane protein-triggered conformation alteration. Proceedings of the National Academy of Sciences 2011, 108:3900–3905. Copyright 2011 by National Academy of Sciences, USA.

By tagging fluorophore directly/non-directly to aptamers, several groups have evaluated aptamer probe accumulation at tumor sites following systemic administration. Shi et al. generated a Cy5-labeled TD05 aptamer, which binds specifically to the IgM heavy chain on Ramos cells. Following tail-vein injection into Ramos tumor-bearing mice, fluorescent signal in non-target tissues faded after 4 hours, while the signal at the tumor site remained noticeable even after 6 hours [49]. The specific aptamer binding promoted prolonged fluorescent signal, enabling the determination of the tumor location. Savla et al. verified that Muc1 aptamer conjugation, allowed QDs to accumulate more specifically at the tumor site than other organs 24 hours following their injection to an A2780/AD ovarian xenograft model [54]. Additionally, an activatable probe has been developed based on the Sgc8 aptamer which targets CCRF-CEM cells. Upon target binding, the probe changes conformation to dissociate the quencher from the fluorophore, resulting in signal restoration. During the *in vivo* study with CCRF-CEM tumor-bearing mice (shown in Figure 3), fluorescence was clearly visible at the tumor site between 15 to 180 minutes. Compared to the “always-on” aptamer probe which yielded higher background signals on non-tumor tissues, the activatable probe was highly promising to significantly enhance imaging contrast and reduce diagnosis time [55].

Simultaneous detection of various cancer cell types is another useful application of aptamer biosensors. This was achieved previously through optical means *in vitro*, by labeling the different cancer cells with distinct aptamer-conjugated fluorescent NPs. Kang et al. conjugated three aptamers: AS1411 (targeting nucleolin), TTA1 (for tenascin-C), and MUC-1 (for mucin) with three QDs of distinct emission (605, 655, and 705 nm) to identify five cancer cell types based on the differential expression of their respective biomarkers [56]. Meanwhile, Chen et al. were able to simultaneously detect Ramos, CEM and Toledo cancer cells from a cell mixture, by applying three FRET NPs modified with distinct aptamer (TD05, Sgc8, and T1, respectively) [57].

As for PET/SPECT monitoring, aptamer conjugation has been used to enable control over the biodistribution of radio-isotope tracers. Previously, aptamers generated against activated neutrophil elastase NX21909 were labeled with ^99m^Tc, to monitor inflammatory events in a rat [58]. In a similar manner, an aptamer against tenascin-c (TTA1) was labeled with Rhodamine red and ^99m^Tc to track glioblastoma (U251) and breast cancer (MDA-MB-436) cells. Following intravenous injection, the aptamer sensors showed rapid accumulation at tumor site within 10 minutes and diffused throughout 3 hours after. The rapid tumor uptake, in conjunction with rapid renal/hepatic clearance, resulted in a high tumor to blood signal ratio (~50 in 3 hours) [59]. Pieve et al. also explored the potential usage of antiMUC1-aptamers as a radiolabelling agent for breast cancer. Selected aptamers which were conjugated with MAG2 ligand and labeled with ^99m^Tc, demonstrated great accumulation at the tumor site of MCF-7 tumor-bearing mice [60]. More recently, conjugation of the A10-3 RNA aptamer with ^64^Cu through several chelators was shown *in vitro* not to compromise A10-3 specificity towards PSMA positive tumor, therefore being another promising candidate for aptamer radiolabeling [61].

For cell tracking via X-ray/CT, findings that AuNP has greater X-ray absorption compared to iodine-based contrast agents with less tissue interference and cytotoxic effects have encouraged aptamer-AuNP probe utilization [62, 63]. Kim et al. developed a bifunctional aptamer AuNP probe for combined prostate cancer imaging and treatment. Particularly, the A9 aptamer was extended to allow conjugation of a Dox loaded-21-nt (GCA)_7_ linker, with thiol modification for subsequent AuNP attachment. The NPs increased CT intensity by 4 folds in PSMA positive LNCaP cells when compared to control PC3 cells, indicating a promising selective CT contrast agent [64]. This report showcases the potential of achieving an integrative aptamer probe to promote drug localization as well.

Similarly, magnetic NP-aptamer conjugates are promising candidates in MRI. For example, Ko et al. conjugated nucleolin aptamer AS1411 to gallium-67 attached magnetic fluorescent cobalt-ferrite NPs. After intravenous injection into tumor-bearing mice, the NPs showed accumulation at tumor site, observable by both scintigraphy and T2-weighted MR. The incorporation of the AS1411 aptamer led to a strong radionuclide signal with increased T2 signal at the tumor region. In contrast, mutant aptamer AS1411mt (with substituted G core nucleotides) was ejected rapidly from the bloodstream with no accumulation at tumor sites, demonstrating the usefulness of specific binding moieties in tumor detection [33]. In other studies of MRI-based cancer cell tracking, aptamers have been conjugated with superparamanetic iron oxide nanoparticles (SPION) to modify the magnetic relaxation time of the specific cancer cells. Wang et al. showed that anti-PSMA A10 aptamer conjugated-SPION significantly decreased both relaxation times (T1 & T2) of target LNCaP cells, as compared to control PC3 cells [42]. Jalalian et al. also showed great enhancement of T1 weighted MR signal in C26 tumor area, when 5TR1 aptamer targeting Mucin 1 SPION complex was used for simultaneous imaging and Epirubicin drug delivery [65].

Aside from the examples mentioned above, there exist more aptamers targeting upregulated receptors on cancer cells, although their utilization for *in vivo* cell tracking is still limited. These include the GS24, J18, AIR-3A and Sgc8 aptamers, which are respectively targeted to well-known receptors namely transferin receptor (TfR), epidermal growth factor receptor (EGFR), interleukin-6 receptor (IL-6R), and tyrosine kinase receptor 7 (PTK 7) [66–69]. Given that these receptors are closely related to the progression of various cancers (e.g. ovarian, hepatocellular carcinoma, and acute lymphoblastic leukemia (ALL) [70–72]), further application of these aptamers as cancer tracking probes *in vivo* should be very exciting. Not only do aptamer probes reveal the tumor location and status, they can also help to hinder tumor growth when utilized in conjunction with therapeutics for effective drug delivery [71, 72].

### Aptamers for tracking stem cells

Besides their applications for cancer cells, aptamers have also been explored in the stem cell field, particularly because of the increasingly important role of stem cells in regenerative medicine. To date, stem cells have been incorporated *in vivo* for both tissue engineering and cell therapy purposes. Seeded on scaffolds, these stem cells can be expanded *in vitro* to achieve functional tissues for later implantation [73–75]. Additionally, these stem cells (without biomaterials) can be solely injected as cell therapy. Mesenchymal stem cells (MSCs) in particular are achieving acclaim for their immunomodulatory properties. Aside from being multi-potent stem cells which can differentiate to several cell types, MSCs can either promote or suppress the inflammatory response [76, 77]. Consequently, they can escape allogeneic rejection, and have been applied to promote wound-healing or in conjunction with graft placement to suppress the graft-versus-host disease (GVHD) [78, 79]. In addition, some have even reported their role in suppressing cancer cell growth [80, 81].

One usage of aptamers for stem cell applications is to assist their isolation from the body. Noting that stem cells exist in such a small fraction (e.g. MSC exists approximately in a ratio of 1:100,000 to bone marrow cells in teenagers) [73], a highly specific and sensitive aptamer recognition platform is useful to generate a homogenous pool of stem cells. Guo et al. managed to isolate adult mesenchymal stem cells (aMSCs) from the bone marrow cell mixture utilizing both aptamer-assisted magnetic sorting and aptamer-assisted fluorescence activated cell sorting (FACS). Due to the lack of specific surface markers of aMSCs, they initially utilized cell-SELEX to isolate aptamers with high binding affinity compared to peripheral blood cells. One aptamer sequence, G8 was chosen for further studies. For the magnetic column fishing experiment, biotinalyted G8 was used to specifically label aMSCs with streptavidin-coated magnetic Dynabeads^®^. Similarly, the G8 aptamer was fluorescein isothiocyanate (FITC)-labeled to enable FACS. This aptamer-enabled separation method resulted in isolation of aMSCs with correct marker staining showing CD29^+^, CD44^+^, CD45^-^, CD90^+^, SLA class I^+^, SLA DQ^-^, and SLA DR^-^ [82]. Subsequently, they applied their apt-magnetic beads labeling for *in vivo* aMSCs tracking. 20 hours post-injection, the labeled cells were observed with MRI to disperse throughout the myocardium layers, in accordance with the presumed vascular distribution area. Moreover, histological examination of the biopsies confirmed the presence of labeled aMSCs at the identified area [83].

Aptamer labeling can alternatively be applied to monitor the differentiation process of stem cells, as shown by Iwagawa et al. [35]. Their selected aptamers (L1-65, L2-2 and L3-3) showed specific binding affinity to mouse embryonic stem cells (mESCs), with low affinity towards differentiated cell lines. During the course of retinoic acid (RA) differentiation, multiple injections of the aptamer showed declining cell affinity with progressive decrease of fluorescent signal. Applying the same principle, stem cells can potentially be incubated and tagged with similar aptamer probes prior to their injection to facilitate colorimetric evaluation of successful cell differentiation.

**Figure 4:**
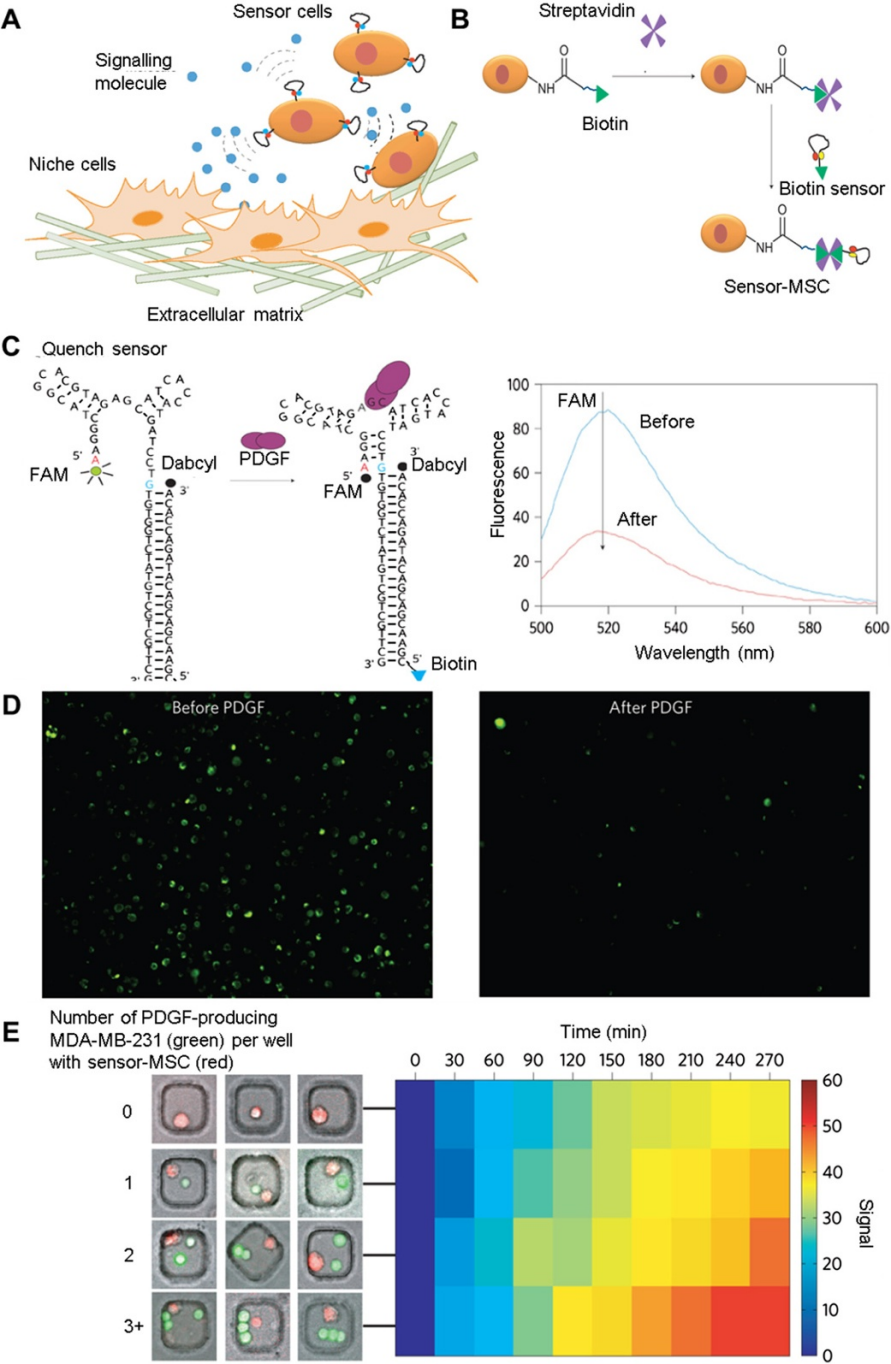
**Aptamer sensor probing stem cells’ niche environment and signalling. A)** Schematic showing the detection of signaling molecules using stem cell-conjugated aptamer probes. **B)** Aptamers were covalently attached to the cell through a streptavidin-biotin conjugation. **C)** Structure of PDGF-sensitive aptamer probes used for this study. Upon specific binding with PDGF, the probe conformation is altered and the fluorescent signal of FAM is quenched by Dabcyl. **D)** Representative fluorescent microscopy images of the sensor-tagged cells before and directly after the addition of 10 nM PDGF in PBS. **E)** Real-time MDA-MB-231 cells’ PDGF secretion monitoring by apt-tagged MSCs. Left: experimental setup with microwells containing one sensor MSC (red) with different numbers of PDGF-producing MDA-MB-231 cells (green). Here the secreted PDGF has been labeled with a GFP tag, while the apt sensor is labeled with a red dye (Cy5). Right: declining MSCs’ fluorescence was observed during the course of PDGF production. The signal, which is defined as the percentage of MSCs with <50% of their initial fluorescent intensity at any indicated time, correlates with the number of MDA-MB-231 cells. Adapted with permission from ref. [50]. Zhao W, Schafer S, Choi J, Yamanaka YJ, Lombardi ML, Bose S, Carlson AL, Phillips JA, Teo W, Droujinine IA: Cell-surface sensors for real-time probing of cellular environments. Nature nanotechnology 2011, 6:524–531. Copyright 2011 by Nature Publishing Group.

Cellular interaction of stem cells with native host cells (e.g. through factor secretion) is another important component which determines the success of regenerative medicine. PDGF for example, is particularly important for MSC signaling, as it participates in MSC communication with activated endothelial cells and recruits them to localize at inflamed tissue or tumors [84, 85]. To this end, Zhao et al. developed an aptamer biosensor to recognize PDGF in the extracellular environment to better understand MSC recruitment and signaling (shown in Figure 4). Facilitated by amine-biotin-streptavidin interactions, PDGF-sensitive aptamer beacons were conjugated onto the surface of MSCs prior to their injection. This aptamer was originally developed and optimized by Tan’s group to specifically recognize PDGF, even in the presence of other proteins in the medium (e.g. bovine serum albumin (BSA), lactate dehydrogenase (LDH)). As the aptamer forms a complex structure upon PDGF binding, it can act as FRET/quench-based sensor that determine nearby PDGF concentration [86, 87]. Through monitoring the aptamer fluorescent signal, the interactions experienced by the MSCs can be gauged, and the cell localization site determined. In this study, the sensor-engineered cells were detected in the BM of the mouse 24 hour post-transplantation [50]. In the future, similar GF-responsive aptamers can even be used to direct the homing of therapeutic cell to a particular diseased site.

### Aptamers for tracking immune cells

Cell-based Immunotherapies are proven to be effective for some diseases such as cancers. One type of immune cells that has gained remarkable attention in the field is the dendritic cells (DCs). DCs are specialized antigen-presenting cells (APCs) with immunostimulatory receptors which aid lymphocyte (T cell) activation. During exposure to infections, immature DCs uptake pathogens, degrade, and display their fragments to trigger further immune responses (also known as DC maturation events) [88, 89]. Exploiting these properties, DCs have been utilized to elicit and control immunoregulatory cell activity, especially in immune-related diseases [90, 91]. To achieve successful cell therapy however, injected DCs need to first migrate to the desired location and survive, before executing their intended cellular function [92, 93]. Furthermore, the process of cell therapy such as tuning of required cell number, injection frequency, and administration strategies can be optimized by continuous tracking [6, 94, 95]. Given their binding specificity and versatility, potential incorporation of aptamers tracking in DC-based therapy will be further discussed below.

**Figure 5:**
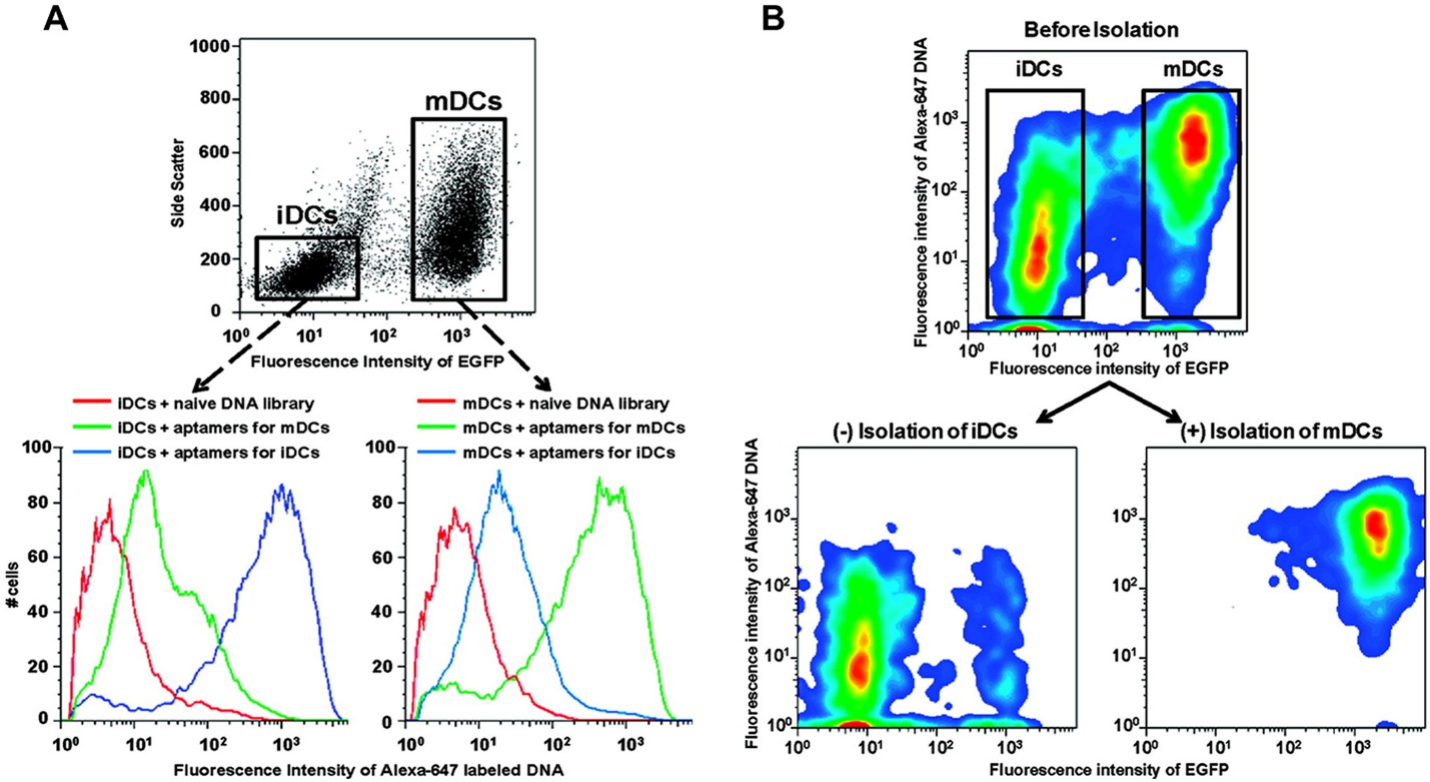
**Aptamer selection for immature (iDCs) and mature dendritic cells (mDCs). A)** Flow cytometry characterization of aptamer pools’ binding to iDCs and EGFP-labeled mDCs after 10 rounds of selection. As evaluated through FC, aptamers selected for one DC type have 100-fold affinity relative to the other DC type. **B)** Aptamer-facilitated mDCs isolation from iDCs with magnetic beads. (top) Flow cytometry assay of EGFP- iDCs and EGFP + mDCs mixture with Alexa-647- and biotin-labeled mDC aptamers. (bottom) Flow cytometry assay following cell isolation with magnetic beads. Cells left in supernatant are predominantly iDCs (left), while cells attached to the beads are mostly mDCs (right). Adapted with permission from ref. [96]. Berezovski MV, Lechmann M, Musheev MU, Mak TW, Krylov SN: Aptamer-facilitated biomarker discovery (AptaBiD). Journal of the American Chemical Society 2008, 130:9137–9143. Copyright 2008 by American Chemical Society.

To date, while there have been no significant reports on aptamer biosensors application to track injected DCs *in vivo*, there are several groups that have applied cell-SELEX technique to identify aptamers with high specificity to DCs. Berezovski et al. for example, carried the selection process to isolate aptamers against immature or mature dendritic cells (iDCs or mDCs), with 100 fold greater affinity against the other DC type. Following which, separation of mDCs from DC mixture was achieved through FACS and streptavidin-coated magnetic beads (shown in Figure 5), by tagging the aptamers with biotin and Alexa-647 fluorophore [96]. Not only do the mDCs aptamers assist *in vitro* DCs preparation (to ensure maturation) prior to their injection, but the aptamer-labeled mDCs can potentially be directed *in vivo* by external magnets, as well as monitored through MRI.

SELEX can also be applied to identify aptamer sequences specific toward known DC ligands. As one example, an aptamer targeting intercellular adhesion molecule (ICAM-3) grabbing non-integrin (DC-SIGN) ligand was selected with high affinity (K_d_ value 21.73 nmol/L) [97]. DC-SIGN has been identified previously as an interesting ligand of DCs, as it allows pathogen binding to escape the immune response [98]. Wengerter et al. have also selected aptamers that recognize DEC205, a C-type lectin which facilitates antigen cross-presentation and CD8+ T cell activation [99]. By conjugating these aptamers with a reporting probe and allowing them to label DCs prior to their injection, the DCs localization can be tracked and evaluated.

Aside from DCs, aptamer applications for immune cells have also aimed at targeting T-cells. Several aptamers have been previously identified for known receptors on activated T cells, including the 4-1BB, OX40, and CTLA-4 receptors [100–102]. Interestingly when introduced *in vivo* as multivalent aptamer structures, the selected aptamers in all three studies were capable of stimulating further T-cell activities and inhibiting tumor growth. Applying similar aptamers but labeled with imaging moieties, subsequent labeling and tracking of the T-cell *in vivo* can elucidate the processes by which the T-cell resides and interferes with tumor growth.

### Challenges and outlook

While aptamers have attracted significant attention in the field of cellular tracking, especially for cancer cells, there are still several considerations that need to be addressed prior to their clinical translation. These include: 1) strategies to enhance *in vivo* pharmacokinetics, 2) loss of *in vivo* affinity for *in vitro* selected aptamers, 3) cost effectiveness of aptamer-based diagnosis, and 4) safety aspect of aptamer biosensors. Below, we will elaborate further on these challenges and propose some solutions. The brief summary of these considerations can be found in Table 1. Lastly, we have also included our perspectives on the future of aptamer technology for cell tracking.

**Table 1:** **Challenges in aptamer biosensors**

Challenges	Possible solutions
*Strategies to enhance in vivo pharmacokinetics*	- Formation of aptamer conjugates
	- Aptamer chemical modifications
Loss of *in vivo* affinity for *in vitro* selected aptamers	- Direct *in vivo* aptamer selection
	- Adjusting *in vitro* selection conditions to mimic closely *in vivo* conditions (e.g. pH, temp)
	- Polyvalent aptamer conjugates
Cost of aptamer biosensors	- Minimizing aptamers length
	- Utilize DNA-based aptamer
Safety of aptamer biosensors	- Complete pre-clinical studies
	- Negative selection with closely related molecular targets

### Strategies to enhance in vivo pharmacokinetics

Since aptamers are small oligonucleotides with average molecular weight between 10 to 15 kDa, they can be subject to rapid nuclease degradation and blood clearance, which significantly affect systemic delivery. Signals from the aptamers (even when bound to their target) can decay rapidly and are therefore not suited for prolonged *in vivo* longitudinal monitoring [103–106]. Meanwhile for some cell tracking applications, cells need to be monitored over an extended period of time, such as stem cell tracking following implantation. To uncover the biodistribution of these cells, the labeling may need to last a few hours. However to further monitor the growth and integration of the stem cells with host tissue, it might be required to track the stem cells for at least a few weeks [107, 108]. In this case, conventional unmodified aptamers would be unsuitable.

To enhance their pharmacokinetic properties, aptamers have previously been complexed with other entities like NPs. The relatively large size of these conjugates, coupled with the multivalent aptamers binding, facilitates faster accumulation at targeted cells and slower elimination from the circulation [109, 110]. Additionally, chemical modifications can be made to prolong the circulation half-life of aptamers. As an example, 40 kDa poly-ethylene-glycol (PEG) molecules have been utilized on the commercially available Macugen aptamer which targets the vascular endothelial growth factors (VEGF) [111]. Modifying the backbone of the aptamers to incorporate protective groups such as 2’-fluoro, 2’-amino, or 2’-O-methyl can also help to improve nuclease resistance. Given that nucleases cause significant aptamer degradation, these protective groups can be utilized to prolong aptamer tracking/labeling window [112, 113]. With these chemical and physical modification tools already available, one can expect significant improvement in the longitudinal cell tracking properties of aptamers over the next few years.

### Loss of in vivo affinity for in vitro selected aptamers

As aptamer target binding involves conformational change that is dependent on external conditions (e.g. pH, temperature, etc.), the great discrepancy between *in vivo* pathophysiological conditions and *in vitro* selection conditions poses a significant issue. The high affinity of the *in vitro* selected aptamers may be negated upon *in vivo* translations [114–116]. One possible solution is to perform the aptamer selection directly *in vivo*, although the complexity may results in the need for more selection rounds [117, 118]. However, *in vitro*-based selection might actually suffice, provided the extracellular conditions are adjusted to closely mimic the *in vivo* conditions [115, 119].

Additionally, conjugating several aptamers together to form multivalent aptamer structures have been shown to assist *in vivo* targeting efficiency [99, 100, 120]. In the study done by Wengerter et al., tetravalent DEC205-aptamer streptavidin (SA) conjugates and not monovalent aptamer-SA were observed to be uptaken by DEC205+ DCs *in vivo*. Furthermore when linked with OVA for T-cell cross presentation, only the tetravalent aptamer construct was capable of eliciting T cell proliferation [99]. As an extension to the multivalency effect of one aptamer type, conjugating multiple aptamers that recognize different receptors/markers of the cells should also greatly enhance aptamer binding affinity. As shown recently, multiple aptamers cell labeling is very possible given that the aptamers do not inhibit each other’s binding [121].

### Cost effectiveness of aptamer diagnostics

Another factor to be considered for the translation of aptamer biosensors is their cost. The unstable nature of RNA oligonucleotides which results in short shelf-life can significantly affect the manufacturing cost of aptamers [122]. Furthermore, large-scale generation of long oligonucleotide sequences can be relatively difficult to achieve via solid-phase-synthesis approaches. Chemical modifications to their backbone also add to the cost of aptamer biosensors [123, 124]. Therefore, while recent synthesis technology advancements have helped to decrease costs, cell-SELEX with shorter sequences should be a viable alternative. Alternatively, sequence minimization can be done following the selection process, to eliminate non-critical regions [115, 125, 126]. In addition, noting that DNA aptamers are relatively more stable compared to RNA aptamers, DNA-based aptamers can be used with less chemical modifications [104, 127].

### Safety of aptamer biosensors

While aptamers have been recognized to be low in immunogenicity compared to most protein-based probes or drugs, the safety aspect of aptamers remain to be carefully examined [128, 129]. Aptamer binding to non-target but similar molecules can trigger the activation of undesired signaling pathways thereby interfering with other aspects of cell status/function [104, 130]. Thus for future clinical translations, rigorous biocompatibility studies need to be performed. Pre-clinical cytotoxicity evaluation should be conducted on various cell types, and a wide variety of pre-clinical animal subjects. Especially when coupled with imaging moieties which can be cytotoxic, preclinical studies should be used to determine the threshold concentration of the aptamer biosensors, at which no adverse side-effects will occur [54]. Concurrently, negative selection with closely related molecular targets should be included within the cell-SELEX process to reduce the potential for non-specific cross-binding [131].

### Perspective: aptamers for cell tracking and assessment of status/functions

With the rapid expansion of cell-SELEX technology recently, it is conceivable that aptamer-based cell tracking will expand significantly in the next couple of years. Aside from the aspects of aptamers mentioned above that need improvement, extending the applications of aptamers in cell tracking can be vital. Applying the concept of aptamer cell tracking in cell therapy to reveal interactions experienced by the injected cells can shed significant light with regards to the therapeutic process. It is commonly known that cellular interactions play a major part in directing the fate of injected cells [132–134]. In the example above, surface-conjugated aptamers were utilized to study PDGF signaling experienced by MSCs [50]. This can be extended to study the effect of various signaling molecules for both stem cells and immune cells therapy (e.g. tumor necrosis factor alpha (TNF-α), VEGF) [135, 136]. Additionally, aptamer has the potential to reveal direct cell-cell communication. By conjugating two aptamers recognizing different cell types for example, their interactions can be studied through aptamer signal dynamics. Ultimately with increased insight into post-transplantation interactions, cell therapy can be greatly optimized. Alternatively, aptamer tracking can be further applied to assist the validation of therapeutic cells *in vitro*. Stem cell culture typically undergoes a number of processing steps prior to usage that may consist of (1) separation from a heterogeneous population or (2) undergo cell transformation (i.e. differentiation) into a heterogeneous population. In contrast, a cell population that bears a (or multiple) homogenous therapeutically relevant biomarker is typically desired in therapy [137, 138]. The selectivity of aptamer-based cellular separation can be utilized to enhance the purity of the implanted cell population. Furthermore, quality assurance can also extend to the identification of undesirable stem cells that generate unwanted teratomas *in vivo* by using suitable aptamers.

## Conclusions

Aptamer-based biosensors selected through cell-SELEX can bring significant improvements to *in vivo* cellular tracking. Aptamers with high binding affinities against specific cancer antigens can be conjugated with various imaging probes to trace the presence of a tumor mass for cancer diagnosis, or with anti-cancer drugs for targeted cancer treatment. In addition to revealing the cancer types based on the detected molecular signature, aptamers provide a means to monitor cancer cells in real time, towards a better understanding of various cancer behaviors. Ultimately, this will lead to greater efficiency in cancer treatment. Furthermore, for therapeutic cell applications, aptamer biosensors can facilitate the monitoring of cellular processes following implantation, while examining the bioactivities of therapeutic cells (e.g. cell differentiation, protein secretion, etc.) can facilitate the optimization process to achieve successful therapy.

Thus far, the reported usage of aptamer sensors for stem and immune cell tracking is still minimal. While many studies have been performed for diseased cancer cell detection and therapy, little emphasis has so far been placed on aptamers for injectable therapeutic cells. Utilizing the cell-SELEX approach however, it is conceivable that aptamer biosensors can be engineered to monitor the process of therapeutic cells specified above (e.g. maturation of DCs) of the therapeutic cells. As shown in previous examples, aptamers highly specific to mature DCs and MSCs have been selected and well applied for their subsequent isolation.

Additionally while limitations in aptamer probe usage remain, including the short circulation lifetime especially for RNA-based aptamer and the difficulty to chemically synthesize long oligonucleotides, these issues may not significantly hinder their implementation. As discussed in the previous section, various modification techniques have been proposed to alleviate these limitations. Modifications to the backbone of aptamers such as 2’-fluoro, 2’-ribo and 2’O-methyl RNA, have been shown to prolong their circulatory lifetime, while conducting cell-SELEX from a pool of short oligonucleotides facilitates simpler chemical synthesis.

In the long run, successful development of aptamer sensors can potentially be adapted into clinical applications and will assist the monitoring of diseased cells from the host body or externally introduced therapeutic cells. At the same time, the versatile nature of aptamer-based probes can be harnessed as well for targeted treatment *in vivo*, thereby realizing the notion of integrated theranostic agents.
